# Sequence and Gene Expression Analysis of Recently Identified NLP from *Plasmopara viticola*

**DOI:** 10.3390/microorganisms9071453

**Published:** 2021-07-06

**Authors:** Lars Askani, Stefan Schumacher, René Fuchs

**Affiliations:** Department of Biology, State Institute of Viticulture and Enology, 79100 Freiburg, Germany; lars.askani@wbi.bwl.de (L.A.); Stefan.Schumacher@wbi.bwl.de (S.S.)

**Keywords:** *Plasmopara viticola*, NLP, necrosis and ethylene inducing peptide 1, grapevine, downy mildew

## Abstract

Grapevine downy mildew, evoked by the obligate biotrophic oomycete *Plasmopara viticola,* is one of the most challenging diseases in viticulture. *P. viticola* establishes an infection by circumvention of plant immunity, which is achieved by the secretion of effector molecules. One family of potential effectors are the necrosis- and ethylene-inducing peptide 1 (Nep1)-like proteins (NLP). NLP are most abundant in plant pathogenic microorganisms and exist in cytotoxic and non-cyctotoxic forms. Cytotoxic NLP often act as virulence factors and are synthesized in necrotrophic or hemibiotrophic pathogens during the transition from biotrophic to necrotrophic growth. In addition to these cytotoxic NLP, many non-cytotoxic NLP have been identified; their function in biotrophic pathogens is still unknown. In 2020, eight different *NLP* coding genes were identified in *P. viticola* and named *PvNLP1* to *PvNLP8* (*Plasmopara viticola* *NLP 1–8*). In the present study, *PvNLP4* to *PvNLP8* were characterized by using qPCR analysis and transient expression in the model plant *Nicotiana benthamiana*. Gene expression analysis showed high *PvNLP* expression during the early stages of infection. Necrosis-inducing activity of *PvNLP* was not observed in the nonhost *N. benthamiana*.

## 1. Introduction

Necrosis- and ethylene-inducing peptide 1 (Nep1)-like proteins (NLP) have been identified in almost 500 different species of bacteria, fungi and oomycetes [1]. Phylogenetic and amino acid sequence analysis revealed conserved patterns that affect the tertiary structure of these proteins and allow for the further division of NLP into three different types [2,3]. With few exceptions, all NLP from oomycetes are type 1 NLP. These NLP share two conserved cysteine residues and can be further divided into type 1 and type 1a by the presence or absence of a cation-binding pocket, respectively. The disulfide bridge between these cysteine residues as well as the cation-binding pocket are essential for the necrosis-inducing ability of these NLP [4,5]. Type 2 NLP, previously observed from fungi and bacteria, were just recently discovered in oomycetes [1]. These NLP share four conserved cysteine residues and, besides the cation-binding pocket, also share a predicted Ca^2+^-binding site. This Ca^2+^-binding pocket is necessary to induce necrosis, while the second disulfide bridge has no influence on the cytotoxicity of these proteins [3]. A third class of NLP, classified as type 3, consists of proteins which are less conserved then the other types but normally share three conserved disulfide bridges and have so far only been identified in ascomycete fungi and bacteria [3]. Another conserved region in NLP is a 24 amino acid peptide (nlp24) which is recognized as a pathogen-associated molecular pattern (PAMP) in several plants and consequently induces common pattern-triggered immunity (PTI) responses [6,7]. However, the responsible receptor complex RLP23-SOBIR1-BAK1 was so far only found in some species of *Brassicaceae* and *Asteraceae* [8]. Even though NLP are widely distributed in prokaryotic and eukaryotic microorganisms, their majority was discovered in plant pathogenic oomycetes and ascomycetous fungi [1]. While bacteria or non-phytopathogenic microorganisms mostly encode only one or two of these proteins, oomycetes have acquired up to 70 throughout gene duplication [1]. NLP are assumed to have different functions, possibly depending on the lifestyle of the pathogen. Cytotoxic NLP, e.g., from the bacterium *Pectobacterium carotovorum* or the fungus *Verticillium dahliae*, can act as virulence factors for their pathogens and are usually expressed during phases of their necrotrophic lifestyle [9,10]. Deletion of these NLP in their hosts clearly reduced their virulence on potato tubers or tomato, respectively. Vice versa, overexpression of a *Fusarium oxysporum* NLP in *Colletotrichum coccodes* resulted in increased virulence of the fungus [11]. Furthermore, in *Colletotrichum* species a correlation of increasing numbers of NLP with a broader host range is assumed [12]. However, inactivation of NLP in *Fusarium oxysporum* or *Magnaporthe oryzae* had no effect on the virulence of these pathogens on coca or rice plants, respectively [13,14]. Besides cytotoxic NLP, a large number of non-cytotoxic NLP with a so far unknown function is known from fungi and oomycetes. The obligate biotrophic downy mildew pathogens *Hyaloperonospora arabidopsidis* (*HaNLP*) and *Plasmopara viticola* (*PvNLP*), for example, have only non-cytotoxic NLP [15,16]. However, characterization of *PvNLP* was so far limited to *PvNLP2* (KC243261), *PvNLP3* (MN720268) and the pseudogene *PvNLP1* (MN564834). In order to fill this gap, further characterization of *PvNLP4*–*PvNLP8* is presented here.

## 2. Materials and Methods

The genes of *PvNLP4* (MT722075), *PvNLP5* (MT722076), *PvNLP6* (MT722077), *PvNLP7* (MT722078) and *PvNLP8* (MT722079) were amplified from an isolate collected in a vineyard in Freiburg (Germany) with the corresponding primers (Table 1) and sequenced as published before [16].

To identify possible sequence differences in *PvNLP* between the different isolates, sequence comparisons were performed with the three available genome assemblies, INRA-PV221 (GenBank: GCA_001695595.3), PvitFEM01 (GenBank: GCA_003123765.1), and JL-7-2 (GenBank: GCA_001974925.1)

To investigate *PvNLP* gene expression during the infection process, experiments were performed as published [16] (pp. 3–4) with the Biozym Blue S’ Green qPCR Kit (Biozym Scientific GmbH, Hessisch Oldendorf, Germany) according to the manufacturer’s instructions and the corresponding primers (Table 2) [16].

Gene expression of *PvNLP4*–*PvNLP8* was monitored over the whole course of infection according to stages defined before [16] (pp.3–4) from 1 to 144 h post inoculation (hpi), and *PvRXLR28* (KX010958) was monitored as expression pattern reference [17].

To study the cytotoxic characteristics of *PvNLP4*–*PvNLP8* the corresponding genes were amplified with the appropriate primers (Table 3) and transiently expressed in *N. benthamiana* as published before [16]. To verify the functionality of the experiments, a necrosis-inducing NLP from *P. infestans* (*Pi*NPP1.1; AY961417) as well as the necrosis-inducing effector RXLR35 (P0CV04) from *P. viticola* were expressed as positive controls [18,19]. Agrobacteria carrying the respective plasmids were co-infiltrated with the silencing suppressor p19 [20]. To exclude an effect of p19, the plasmid was infiltrated alone as negative control.

## 3. Results & Discussion

### 3.1. Sequence Analysis of PvNLP4–PvNLP8

Sequence analysis of the genes *PvNLP4* (MT722075), *PvNLP5* (MT722076), *PvNLP6* (MT722077), *PvNLP7* (MT722078) and *PvNLP8* (MT722079) showed several differences compared to the genes inside the reference genomes INRA-PV221 (GenBank: GCA_001695595.3), PvitFEM01 (GenBank: GCA_003123765.1), and JL-7-2 (GenBank: GCA_001974925.1). *PvNLP4* shows one silent point mutation (g^159^ → c^159^). *PvNLP5* is identical to the corresponding sequences in INRA-PV221 and PvitFEM01, but carries the silent mutation c^48^ → t^48^ compared to JL-7-2. *PvNLP6* was only identified in INRA-PV221 [16]. The gene sequenced during this work carries 12 point mutations: t^48^ → c^48^, c^52^ → g^52^, c^216^ → t^216^, t^273^ → c^273^, c^372^ → t^372^, a^399^ → t^399^, t^400^ → a^400^, a^427^ → g^427^, a^448^ → g^448^, a^458^ → t^458^, c^654^ → t^654^ and a^657^ → g^657^. While eight mutations are silent, five result in the amino acid substitutions R^18^ → G^18^, L^134^ → I^134^, I^143^ → V^143^, K^150^ → E^150^, D^153^ → V^153^. Only two of these alterations, L^134^ → I^134^ and I^143^ → V^143^, retain the nonpolar and hydrophobic properties of the original amino acids. The amino acid substitution L^134^ → I^134^ is located inside the GHRHDWE heptapeptide motif where G^134^ is replaced with L^134^ in INRA-PV221 [16]. Type 1 NLP have been divided into type 1 NLP and non-cytotoxic type 1a NLP based on the occurrence of amino acid substitutions in the GHRHDWE heptapeptide motif [3]. The heptapeptide motif is part of a negatively charged cavity implicated in cation binding (Mg^2+^) and a specific interaction with the sphingolipid glycosyl inositol phosphorylceramide (GIPC) [5,21]. Therefore, the heptapeptide motif is essential for the cytotoxic activity of NLP [5]. However, the heptapeptide motif is prone to mutations in type 1a NLP, such as *PvNLP*6, and probably not essential for any additional function other than necrosis induction [3]. The amino acid substitution I^143^ → V^143^ in *PvNLP6* is also highly conserved within the central region of the NPP1 (necrosis-inducing *Phytophthora* protein) domain [2]. Gijzen and Nürnberger [2] (p. 1802) highlighted the alterations of the first five amino acids following the heptapeptide motif. The third of these five amino acids corresponds to V^143^ of *PvNLP6*. In the majority of all analyzed sequences a valine was conserved at this position, followed by alanine as the second and isoleucine as the third most conserved amino acid [2]. The amino acid substitutions K^150^ → E^150^ and D^153^ → V^153^ alter the chemical properties of the protein and may therefore have a direct influence on the tertiary structure and the functionality of the protein. However, both amino acids are located in a non-conserved region of type 1 / 1a NLP, which was so far not directly linked to any function [3]. The substitution R^18^ → G^18^ is located inside the signal peptide. Since the signal peptide is cleaved off after the secretion process, it has no direct effect on the functionality of the protein. The D-score predicted by SignalP is affected only marginally and changes from 0.8795 to 0.8449 [16] (p.5), therefore this substitution was also assumed as uncritical.

*PvNLP7* was identified in INRA-PV221 and JL-7-2 [16]. The *PvNLP7* sequenced in this study is identical to the corresponding sequence in INRA-PV221 but shows the seven mutations a^112^ → t^112^, g^237^ → a^237^, a^375^ → t^375^, t^492^ → c^492^, t^525^ → c^525^, c^702^ → t^702^ and c^723^ → t^723^ in J-L-7-2. Six of these mutations are silent while one results in the amino acid substitution T^112^ → S^112^. This change in *PvNLP*7 involves two neutral amino acids with hydrophilic characteristics and its influence on the function of the protein is assumed as low. The *PvNLP8* amplified during this study is identical to the sequences from INRA-PV221 and PvitFEM01 and was not identified in JL-7-2 [16]. In summary, all genes could be amplified in the present field isolate. Previous work showed that none of the three reference genomes contains all eight *PvNLP* [16]. However, it is not clear if this divergence is isolate dependent or caused by technical issues during assembling of the reference genomes.

### 3.2. Gene Expression Analysis

**Figure 1:**
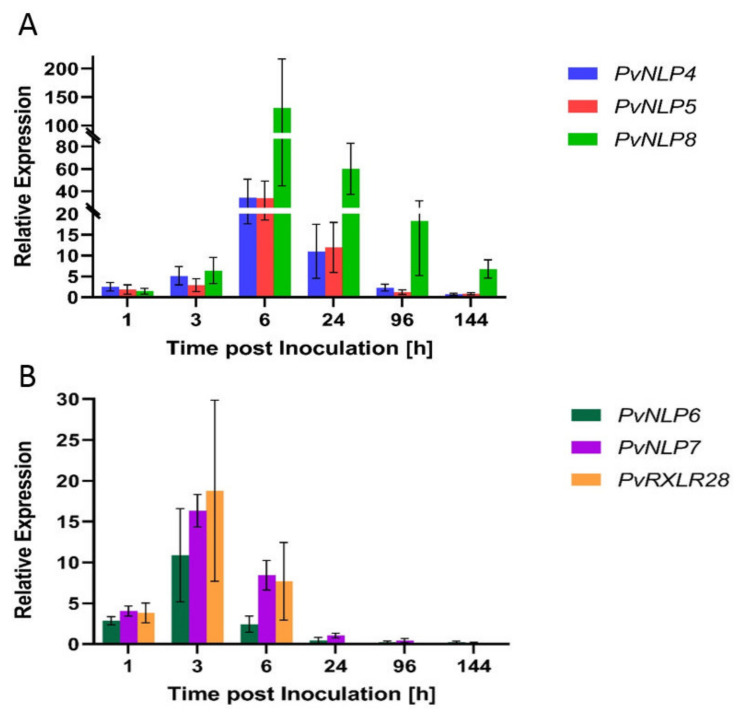
Expression of *PvNLP* during infection of *Vitis vinifera* cv. Mueller-Thurgau leaf cells: (**A**) Relative expression of *PvNLP4*, *PvNLP5* and *PvNLP8*. (**B**) Relative expression of *PvNLP6*, *PvNLP7* and *PvRXLR28*. The displayed graphs represent one of four experiments with similar results. Bars are the mean of four technical replicates ± standard deviation (SD).

All analyzed genes were activated shortly after contact of sporangia with water (Figure 1). *PvNLP4* and *PvNLP5* showed increased expression levels over the first 24 hpi. Levels increased from this time point on, when germ tubes entered the leaf (3 hpi), and the maximum expression level of both genes was reached during the formation of first haustoria (6 hpi). Thereafter, the expression levels decreased during branching of hyphae (24 hpi) to a not detectable level. This expression pattern was also detected for *PvNLP8*, the strongest induced gene during this study. However, the expression of *PvNLP8* stayed elevated when *P. viticola* begins to infect adjacent interveinal leaf zones (96 hpi) until *de novo* formation of sporangia (144 hpi). Expression over the whole course of infection was also observed for *PvNLP2* and *PvNLP3* suggesting a different function of these proteins not only at the beginning of the infection [16]. By contrast, *PvNLP6* and *PvNLP7* as well as *PvRXLR28,* are expressed during the first six hours after inoculation. Expression starts with the release of zoospores from sporangia (1 hpi) and culminates when germ tubes enter the leaf (3 hpi). Once the formation of first haustoria occurs (6 hpi), expression decreases to a not detectable level. *PvRXLR28*, which served as reference for early induced effectors [17] (p.4), showed highest expression levels 3 hpi. This time point was not analyzed in the original work but *PvRXLR28* is described to be expressed during the first 12 hpi, showing an expression peak at 6 hpi and declining afterwards to an undetectable level at 24 hpi [17]. This change in gene expression was also observed in this study, indicating a similar expression pattern in the isolate used in this study and in the Chinese isolate ZJ-1-1.

### 3.3. Necrosis-Inducing Activity

**Figure 2:**
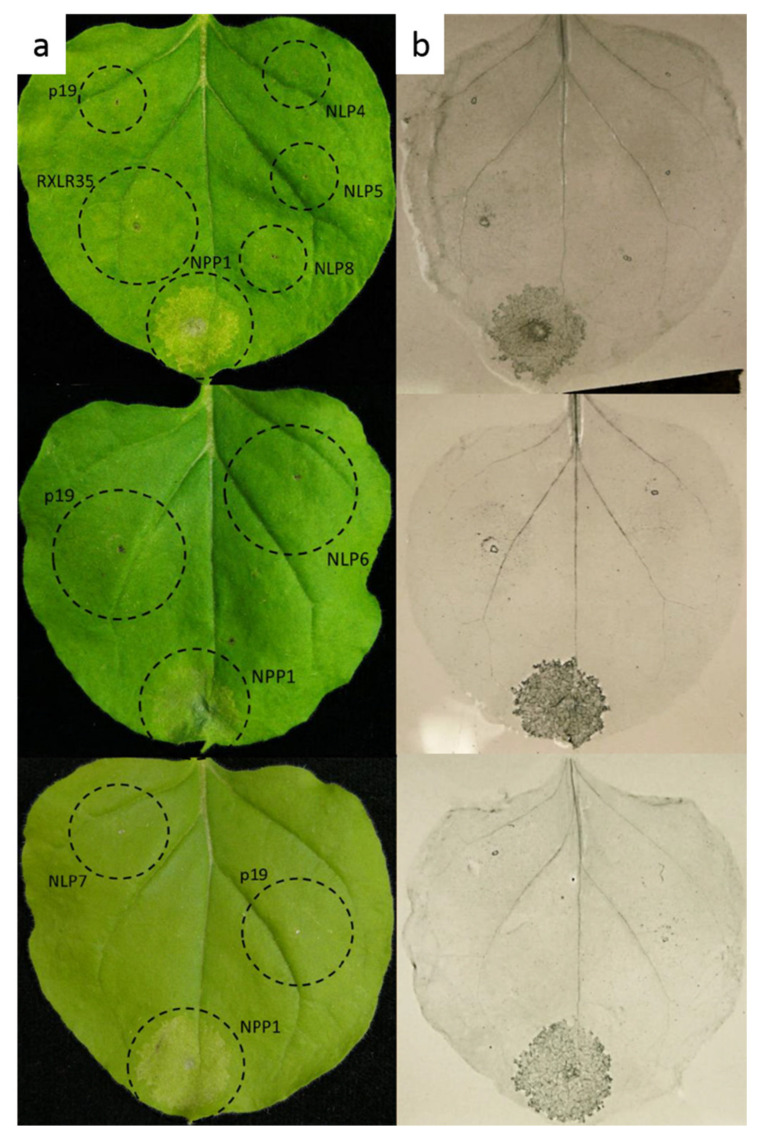
*PvNLP* are not able to induce necrosis in *N. benthamiana*: (**a**) Transient expression of NLP in leaves of *N. benthamiana*; (**b**) Trypan Blue staining for cell death. All pictures were taken 72 h post infiltration. (p19 = silencing suppressor; NPP1 = *Pi*NPP1.1; RXLR35 = *Pv*RXLR35, NLP (4–8) = *PvNLP*4–8).

Cytotoxic characteristics of *PvNLP*4-*PvNLP*8 were studied by agrobacterium-mediated transient expression in *N. benthamiana*. None of the five *PvNLP* (*PvNLP*4, *PvNLP*5, *PvNLP*6, *PvNLP*7 or *PvNLP*8) were able to induce necrosis in *N. benthamiana* (Figure 2). Spots infiltrated with the positive control *Pi*NPP1.1 and *Pv*RXLR35 showed beginning chlorosis after 48 h, which developed into necrotic tissue within the next 24 h. Trypan blue staining was performed at 72 h post infiltration to visualize cell death, indicating beginning necrosis formation on the infiltration sites of *Pi*NPP1.1 and *Pv*RXLR35. Infiltration sites with *PvNLP* were observed for a period of 14 days. During this time no necrosis formation occurred. None of the *PvNLP* or p19 infiltration sites showed a HR with trypan blue staining. The results are in line with the previous published results [16] (p.10) for *PvNLP*1, *PvNLP*2 and *PvNLP*3, which were also not able to induce necrosis. It should be considered that transient gene expression in tobacco is a non-host-specific method to determine the cytotoxicity of an NLP. However, many NLP were analyzed by transient expression in tobacco in the past, and this method was suggested as highly efficient for this purpose [1,2,3]. Thus, this experiment shows that all analyzed *PvNLP* that were predicted to be non-cytotoxic are in fact not capable of causing necrosis in plants. Taken together, the exact role of non-cytotoxic NLP remains unclear and further biochemical, and structural studies (e.g., the determination of their crystal structures, Co-immunoprecipitations and binding assays with different substrates) are needed to determine the molecular functions of noncytotoxic NLP [1].

## Figures and Tables

**Table 1:** Oligonucleotides used for sequencing *PvNLP* genes.

Primer Name	Sequence (5′→3′)
PvNLP4-ORF-F	ATGTCGCGGACCATGCAATC
PvNLP4-ORF-R	TTAAAAAGGGTACGATTCGTGG
PvNLP5-ORF-F	ATGCGCGTCCTAGTATTGTGTG
PvNLP5-ORF-R	TTAGAAGGGTCGCGCTTTCTC
PvNLP6-ORF-F	ATGCGCACCACGAGCCCATACTC
PvNLP6-ORF-R	TTATCGCACACTTTTCGAGGG
PvNLP7-ORF-F	ATGCACCTTTGTGCTCTTCTC
PvNLP7-ORF-R	TTAAAACGGATACGCCTCCTTC
PvNLP8-ORF-F	ATGCACTTGACAGTCTTTTAC
PvNLP8-ORF-R	TTAGAATGGATAGGATTTACGAAG

**Table 2:** Oligonucleotides used for qPCR.

Primer Name	Sequence (5′→3′)
PvNLP4-qPCR-F	CAACCATTCCTTGTTTCTCCTGCTAC
PvNLP4-qPCR-R	GAAGGGCGATCTCGTTAATTTGGC
PvNLP5-qPCR-F	TCCTAGTATTGTGTGCCGCTGTC
PvNLP5-qPCR-R	GTGGCTTGAACATCAGTGCGA
PvNLP6-qPCR-F	AGCCCATACTCGCACTGTTCTC
PvNLP6-qPCR-R	CAGCGGCTTATACTTGACGGC
PvNLP7-qPCR-F	TCAAAGCACACAAAGCTCAAGTCG
PvNLP7-qPCR-R	GTCAAGTGTCAAAGCAGTCTGTGC
PvNLP8-qPCR-F	TGTCCCACCACTACTTGACTGC
PvNLP8-qPCR-R	GATAGGATTTACGAAGAGCGCGGAA
PvRXLR28-qPCR-F	AGTAACCAGCCCTTACAGACTCC
PvRXLR28-qPCR-R	CTCGTTTGGACTTTGCTCACCTTC

**Table 3:** Oligonucleotides used for cloning binary vectors for transient expression in *N. benthamiana*.

Primer Name	Sequence (5′→3′)
XhoI-PvNLP 4–EcoRI-F	AACTCGAGATGTCGCGGACCATGCAATC
XhoI-PvNLP 4–EcoRI -R	AAAGAATTCTTAAAAAGGGTACGATTCGTGG
XhoI-PvNLP 5–EcoRI-F	AACTCGAGATGCGCGTCCTAGTATTGTGTG
XhoI-PvNLP 5–EcoRI-R	AAAGAATTCTTAGAAGGGTCGCGCTTTCTC
XhoI-PvNLP 6–HindIII-F	AACTCGAGATGCGCACCACGAGCCCATACTC
XhoI-PvNLP 6–HindIII-R	CCCAAGCTTTTATCGCACACTTTTCGAGGG
NotI-PvNLP 7–XhoI-F	AATGCGGCCGCATGCACCTTTGTGCTCTTCTC
NotI-PvNLP 7–EcoRI-R	AAAGAATTCTTAAAACGGATACGCCTCCTTC
NotI-PvNLP 8–XhoI-F	AATGCGGCCGCATGCACTTGACAGTCTTTTAC
NotI-PvNLP 8 –EcoRI-R	AAAGAATTCTTAGAATGGATAGGATTTACGAAG
XhoI-PvRXLR35–EcoRI-F	AACTCGAGATGCGTGGTGCGTATTACATC
XhoI-PvRXLR35–EcoRI-R	AAAGAATTCTTACGAAGCTTTGTCAGTCCTT

## Data Availability

The sequencing data has been uploaded to GenBank. DNA sequences of *PvNLP4*, *PvNLP5*, *PvNLP6*, *PvNLP7* and *PvNLP8* can be found using accession numbers MT722075-MT722079.

## References

[1] Seidl M.F., Van Den Ackerveken G. (2019). Activity and Phylogenetics of the Broadly Occurring Family of Microbial Nep1-like Proteins. Annu. Rev. Phytopathol..

[2] Gijzen M., Nürnberger T. (2006). Nep1-like Proteins from Plant Pathogens: Recruitment and Diversification of the NPP1 Domain across Taxa. Phytochemistry.

[3] Oome S., Van den Ackerveken G. (2014). Comparative and Functional Analysis of the Widely Occurring Family of Nep1-Like Proteins. Mol. Plant-Microbe Interact..

[4] Fellbrich G., Romanski A. (2002). NPP1, a *Phytophthora*-Associated Trigger of Plant Defense in Parsley and Arabidopsis. Plant J..

[5] Ottmann C., Luberacki B. (2009). A Common Toxin Fold Mediates Microbial Attack and Plant Defense. Proc. Natl. Acad. Sci. USA.

[6] Böhm H., Albert I. (2014). A Conserved Peptide Pattern from a Widespread Microbial Virulence Factor Triggers Pattern-Induced Immunity in *Arabidopsis*. PLoS Pathog..

[7] Oome S., Raaymakers T.M. (2014). Nep1-like Proteins from Three Kingdoms of Life Act as a Microbe-Associated Molecular Pattern in *Arabidopsis*. Proc. Natl. Acad. Sci. USA.

[8] Albert I., Böhm H. (2015). An RLP23-SOBIR1-BAK1 Complex Mediates NLP-Triggered Immunity. Nat. Plants.

[9] Mattinen L., Tshuikina M. (2004). Identification and Characterization of Nip, Necrosis-Inducing Virulence Protein of *Erwinia Carotovora* Subsp. Carotovora. Mol. Plant-Microbe Interact..

[10] Santhanam P., Van Esse H.P. (2013). Evidence for Functional Diversification Within a Fungal NEP1-Like Protein Family. Mol. Plant-Microbe Interact..

[11] Amsellem Z., Cohen B.A. (2002). Engineering Hypervirulence in a Mycoherbicidal Fungus for Efficient Weed Control. Nat. Biotechnol..

[12] Baroncelli R., Buchvaldt Amby D. (2016). Gene Family Expansions and Contractions Are Associated with Host Range in Plant Pathogens of the Genus Colletotrichum. BMC Genom..

[13] Bailey B.A., Apel-Birkhold P.C. (2002). Expression of NEP1 by *Fusarium Oxysporum* f. Sp. *Erythroxyli* after Gene Replacement and Overexpression Using Polyethylene Glycol-Mediated Transformation. Phytopathology.

[14] Fang Y.L., Peng Y.L. (2017). The Nep1-like Protein Family of *Magnaporthe Oryzae* Is Dispensable for the Infection of Rice Plants. Sci. Rep..

[15] Cabral A., Oome S. (2012). Nontoxic Nep1-Like Proteins of the Downy Mildew Pathogen *Hyaloperonospora Arabidopsidis*: Repression of Necrosis-Inducing Activity by a Surface-Exposed Region. Mol. Plant-Microbe Interact..

[16] Schumacher S., Grosser K. (2020). Identification and Characterization of Nep1-Like Proteins from the Grapevine Downy Mildew Pathogen *Plasmopara Viticola*. Front. Plant Sci..

[17] Xiang J., Li X. (2016). Studying the Mechanism of *Plasmopara Viticola* RxLR Effectors on Suppressing Plant Immunity. Front. Microbiol..

[18] Kanneganti T.-D., Huitema E. (2006). Synergistic Interactions of the Plant Cell Death Pathways Induced by *Phytophthora Infestans* Nep1-Like Protein PiNPP1.1 and INF1 Elicitin. Mol. Plant-Microbe Interact..

[19] Liu Y., Lan X. (2018). In Planta Functional Analysis and Subcellular Localization of the Oomycete Pathogen *Plasmopara Viticola* Candidate RXLR Effector Repertoire. Front. Plant Sci..

[20] Lakatos L., Szittya G. (2004). Molecular Mechanism of RNA Silencing Suppression Mediated by P19 Protein of Tombusviruses. EMBO J..

[21] Lenarčič T., Hodnik V. (2017). Eudicot Plant-Specific Sphingolipids Determine Host Selectivity of Microbial NLP Cytolysins. Science.

